# Case Report: Single-incision laparoscopic sleeve gastrectomy plus jejunojejunal bypass for the treatment of type 2 diabetes in patients with obesity: a case series and review

**DOI:** 10.3389/fsurg.2026.1855934

**Published:** 2026-06-11

**Authors:** Haobo Gou, Qiuya Wei, Jiaxuan Wang, Tianyu Gao, Shibo Lian, Linlin Hou, Minxue Chen, Yong Fan

**Affiliations:** 1 Department of General Surgery, Lanzhou University Second Hospital, Lanzhou University Second Clinical Medical College, Lanzhou, China; 2 NHC Key Laboratory of Diagnosis and Therapy of Gastrointestinal Tumor, Gansu Provincial Hospital, Lanzhou, China

**Keywords:** metabolic syndrome, severe obesity, single-incision laparoscopy, sleeve gastrectomy plus jejunojejunal bypass, T2DM

## Abstract

**Background:**

Single-incision laparoscopic sleeve gastrectomy plus jejunojejunal bypass (SILSG-JJB) is a novel modification of metabolic bariatric surgery. Clinical experience with this approach remains limited, particularly in patients with severe obesity and type 2 diabetes mellitus (T2DM).

**Case presentation:**

We present a case series of three female patients aged 23–30 years with severe obesity complicated by metabolic syndrome and T2DM who underwent SILSG-JJB. Perioperative course and short-term metabolic outcomes were recorded. All procedures were successfully completed through a single incision without additional port placement or conversion to open surgery. No major intraoperative or perioperative complications occurred. Postoperative recovery was uneventful, and all patients were discharged on postoperative day 3. During the short-term follow-up period, satisfactory weight loss and improvement in fasting blood glucose levels were observed, with no procedure-related adverse events or postoperative malnutrition.

**Conclusions:**

This case series demonstrates the technical feasibility and short-term safety of SILSG-JJB in selected patients with severe obesity and T2DM. Short-term follow-up indicated that SILSG-JJB could achieve significant weight reduction and improve metabolic comorbidity-related parameters, including those associated with T2DM. However, its long-term efficacy and safety in obese patients with T2DM and other metabolic complications remain to be further investigated. Further multicenter studies with larger cohorts and longer follow-up durations are required to determine its long-term efficacy, safety, and generalizability.

## 1 Introduction

**Figure 1:**
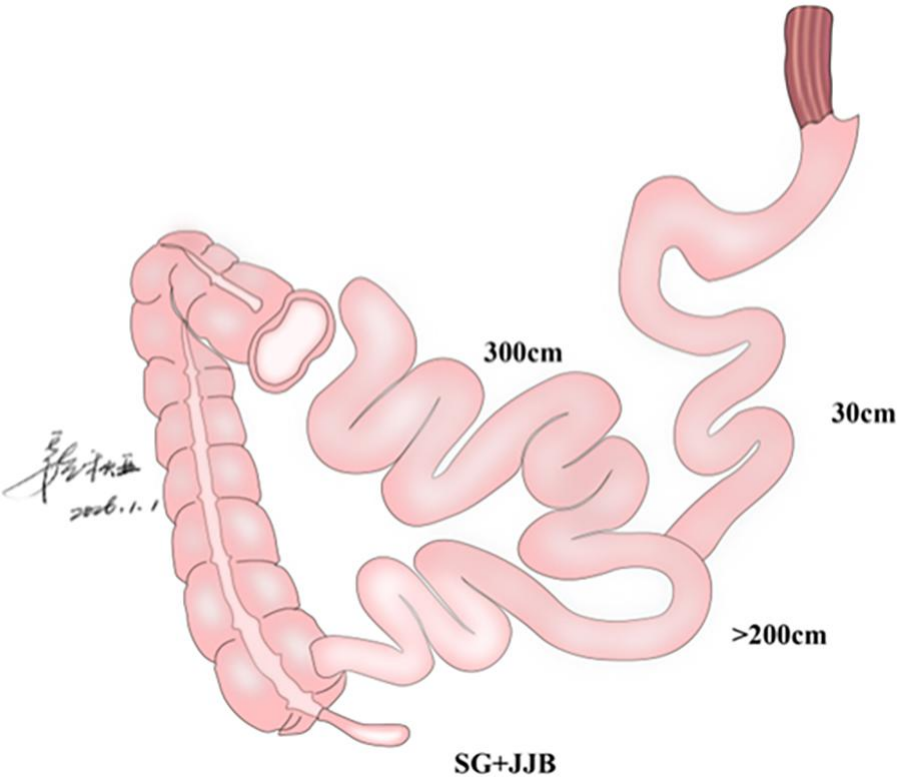
Schematic illustration of the SG-JJB procedure showing a 30 cm jejunojejunal anastomosis from the proximal small intestine, an approximately 300 cm bypassed segment, and a common channel exceeding 200 cm to the ileocecal junction.

According to a 2022 report by the World Health Organization, the global number of individuals with obesity (defined as a body mass index (BMI) ≥30 kg/m^2^) has exceeded 1.083 billion (1). The prevalence of obesity among Chinese adults has reached 16.4%, and obesity-related metabolic disorders are associated with a substantially increased risk of morbidity and mortality, ranking among the leading contributors to disease burden in China (2). Metabolic and bariatric surgery (MBS) represents an important therapeutic approach for obesity, leading to significant improvements in clinical outcomes and quality of life, as well as a reduction in obesity-related mortality (3). According to data from the American Society for Metabolic and Bariatric Surgery (ASMBS), approximately 280,000 MBS procedures were performed in the United States in 2022 (4). Over more than 70 years of development, MBS has evolved into several established surgical procedures, including sleeve gastrectomy (SG), Roux-en-Y gastric bypass (RYGB), sleeve gastrectomy plus jejunojejunal bypass (SG-JJB), and sleeve gastrectomy with transit bipartition (SG-TB) (5). In recent years, advances in minimally invasive techniques, together with increasing patient concern regarding cosmetic outcomes, have led to growing interest in single-incision laparoscopic metabolic and bariatric surgery(SILMBS). This trend is particularly evident in China, where patients undergoing MBS are predominantly female (>70%) and relatively young, with a mean age of 32–34 years; women and younger patients tend to express a stronger preference for SILMBS (6). However, the application of single-incision laparoscopy to complex bariatric procedures, especially SG-JJB, remains challenging because of limited operative space and the complex anatomy associated with obesity. Consequently, data regarding the safety and feasibility of this approach are still scarce. In this study, we report a case series of SILSG-JJB procedures performed at our institution (Figure 1), aiming to explore the clinical applicability of this technique in selected patients with obesity.

## 2 Case presentation

### 2.1 Case 1

**Figure 2:**
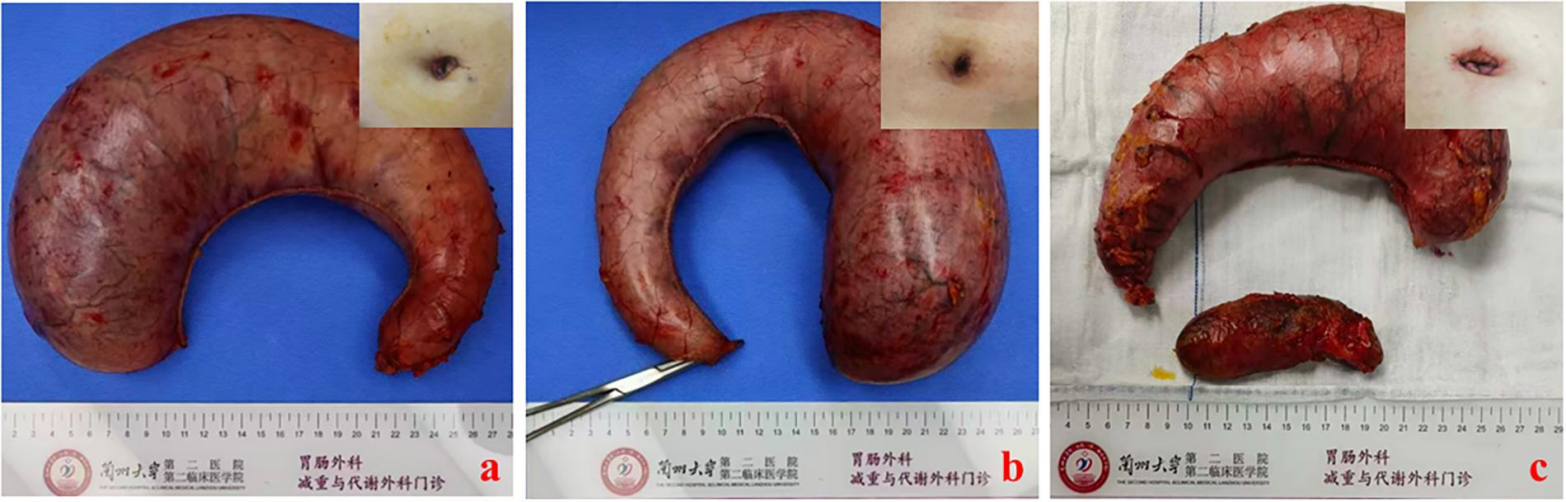
**(a,b)** resected specimens from the greater curvature in cases 1 and 2, and the transumbilical mini-incision at postoperative day 3. **(c)** Resected specimens from the greater curvature and gallbladder in Case 3, and the transumbilical mini-incision at postoperative day 3.

A 23-year-old woman was referred to the metabolic and bariatric surgery clinic because of progressive weight gain. She had undergone laparoscopic cholecystectomy 7 years earlier and had no previous diagnosis of hypertension or diabetes. Her medical history included occasional fasting acid reflux, heartburn, and nocturnal snoring, without evidence of obstructive sleep apnea. At presentation, her body weight was 105 kg and height was 160 cm, corresponding to a body mass index (BMI) of 41.0 kg/m^2^, meeting the criteria for obesity III. The 2-h plasma glucose level during a 75 g oral glucose tolerance test was 13.20 mmol/L, leading to a diagnosis of type 2 diabetes mellitus (T2DM) (7).Laboratory evaluation, including biochemical and insulin-related parameters (Table 1), indicated metabolic syndrome, hyperinsulinemia, insulin resistance, and vitamin D deficiency. Cranial magnetic resonance imaging, adrenal computed tomography, and upper gastrointestinal endoscopy were performed to exclude secondary causes of obesity and gastroesophageal reflux disease. After multidisciplinary evaluation by the metabolic bariatric team, including endocrinology, anesthesiology, respiratory medicine, cardiology, otolaryngology, gastroenterology, and nutrition specialists, the patient was deemed to have normal cardiopulmonary function and to meet the indications for bariatric surgery. Considering surgical safety, enhanced recovery, and cosmetic outcomes, SILSG-JJB was selected. The procedure was completed successfully, with an operative time of 150 min and an intraoperative blood loss of 15 mL (Table 2).The patient passed flatus on postoperative day 2 and was discharged on postoperative day 3 (Figure 2a), with a daily oral fluid intake exceeding 1,500 mL.

**Table 1:** Abnormal laboratory findings in three cases.

Parameter	Case 1	Case 2	Case 3	Reference range	Unit
FPG	5.28 (N)	7.93 (H)	5.22 (N)	3.9–6.1	mmol/L
2h-OGTT	13.20 (H)	12.13 (H)	9.86 (H)	3.9–7.8	mmol/L
HbA1c	6.8 (H)	7.4 (H)	6.2 (H)	4.0–6.0	%
FINS	61.48 (H)	51.25 (H)	25.16 (H)	3.00–25.00	mU/L
TC	6.71 (H)	6.35 (H)	4.21 (N)	2.3–5.2	mmol/L
TG	1.82 (H)	7.53 (H)	1.36 (N)	0.56–1.70	mmol/L
LDL-C	4.62 (H)	2.89 (N)	2.92 (N)	1.20–3.30	mmol/L
UA	448.0 (H)	318.6 (N)	406.1 (H)	155–357	μmol/L
25(OH)Vit D	12.20 (L)	13.90 (L)	24.60 (L)	30–100	ng/mL

FPG, fasting plasma glucose; 2h-OGTT, 2 h oral glucose tolerance test; HbA1c, glycated hemoglobin; FINS, fasting insulin; TC, total cholesterol; TG, triglycerides; LDL-C, low-density lipoprotein cholesterol; UA, uric acid; 25(OH)D, 25-hydroxyvitamin D; N, normal; H, high; L, low.

**Table 2:** Perioperative indexes of patients.

Indexes	Case1	Case2	Case3
Duration of surgery (min)	150	130	170
Intraoperative blood loss (mL)	15	15	20
exhaust time (h)	40	36	38
Length of postoperative hospital stay (d)	3	3	3

Intraoperative blood loss is measured by counting the gauze pads used during surgery.

According to the *Chinese Clinical Guidelines for the Surgery of Obesity and Metabolic Disorders (2024 Edition)*, all patients undergoing bariatric surgery at our bariatric and metabolic surgery center routinely received postoperative supplementation with isolated whey protein and multivitamin chewable tablets containing vitamin A (3,000 μg), vitamin B1 (36 mg), vitamin B12 (1,000 μg), vitamin D3 (75 μg), vitamin E (100.5 mg), vitamin K (300 μg), iron (45 mg), folic acid (800 μg), zinc (16 mg), and copper (2 mg).Short-term postoperative follow-up was conducted at 1 and 3 months after surgery. Follow-up assessments included comprehensive biochemical testing, complete blood count, anemia-related indicators, and serum 25-hydroxyvitamin D measurement. At the 3-month follow-up, no surgery-related complications or nutritional deficiencies were observed (Supplementary Tables S1, S2). In addition, vitamin D deficiency showed significant improvement. Her body weight decreased from 105 kg preoperatively to 91.5 kg at 1 month and 80.0 kg at 3 months after surgery, corresponding to increases in %TBWL from 12.9% to 23.8% and %EWL from 32.9% to 61.0% (Table 3). And glycemic parameters had normalized, with fasting blood glucose levels below 6.1 mmol/L, without the use of antidiabetic medications.

**Table 3:** Postoperative weight loss outcomes.

Variable	Case 1	Case 2	Case 3	Mean
Height (m)	1.60	1.55	1.64	1.60
Preoperative weight (kg)	105	100	120	108.3
Preoperative BMI (kg/m^2^)	41.0	41.6	44.6	42.4
Ideal body weight (kg)	64.0	60.1	67.2	63.8
1 month after surgery				
Weight (kg)	91.5	87.0	107.5	95.3
%TBWL	12.9	13.0	10.4	12.1
%EWL	32.9	32.5	23.7	29.7
%EBMIL	33.0	32.6	23.7	29.8
3 months after surgery				
Weight (kg)	80.0	78.0	97.0	85.0
%TBWL	23.8	22.0	19.2	21.7
%EWL	61.0	55.0	43.6	53.2
%EBMIL	61.1	55.1	43.6	53.3

BMI, body mass index; %TBWL, percentage of total body weight loss; %EWL, percentage of excess weight loss; %EBMIL, percentage of excess BMI loss. Ideal body weight was calculated assuming a BMI of 25 kg/m^2^.

### 2.2 Case 2

A 30-year-old woman presented with progressive weight gain accompanied by metabolic abnormalities for approximately 17 years. With increasing body weight, she developed nocturnal snoring with episodes of awakening due to choking, as well as symptoms of gastroesophageal reflux, including acid regurgitation and heartburn. However, preoperative gastroduodenoscopy and chest CT examinations revealed neither reflux esophagitis nor hiatal hernia. In addition, the patient's reflux symptoms mainly occurred after excessive alcohol consumption; therefore, the patient was not diagnosed with gastroesophageal reflux disease (GERD). She had previously attempted weight loss through dietary modification and physical exercise on multiple occasions, without sustained success. She had no prior diagnosis of T2DM. At presentation, her body weight was 100 kg and height was 155 cm, corresponding to BMI of 41.6 kg/m^2^, meeting the criteria for obesity III (8). The 2-h plasma glucose level during a 75 g oral glucose tolerance test was 12.13 mmol/L, leading to a diagnosis of T2DM. Laboratory findings (Table 1) indicated metabolic syndrome, T2DM, hyperinsulinemia, insulin resistance, and vitamin D deficiency. Following multidisciplinary team (MDT) evaluation, the patient was considered to have clear indications for bariatric surgery and no surgical contraindications. Given her BMI > 40 kg/m^2^ in combination with T2DM, SILSG-JJB was selected. The procedure was completed successfully, with an operative time of 130 min and an intraoperative blood loss of 15 mL (Table 2). The postoperative course was uneventful. The patient reported minimal incisional pain, with no signs of erythema or swelling, and was discharged on postoperative day 3 (Figure 2b). Postoperatively, routine supplementation with isolated whey protein and multivitamin chewable tablets was administered. During the short-term follow-up at 1 and 3 months, no evidence of malnutrition was observed. Serum 25-hydroxyvitamin D levels improved, although they had not fully returned to the normal range. In addition, no surgery-related complications, including postoperative bleeding or anastomotic leakage, were identified during the short-term follow-up period (Supplementary Tables S1, S2). Her body weight declined from 100 kg before surgery to 87.0 kg at 1 month and further to 78.0 kg at 3 months, with %TBWL increasing from 13.0% to 22.0% and %EWL from 32.5% to 55.0% (Table 3), and glycemic levels had normalized without the use of antidiabetic medications.

### 2.3 Case 3

A 30-year-old woman with a body weight of 120 kg and height of 164 cm was evaluated for obesity-related concerns persisting for more than 3 years. Her BMI was 44.6 kg/m^2^, meeting the criteria for severe obesity. Following a 75 g oral glucose tolerance test and comprehensive biochemical evaluation (Table 1), she was diagnosed with metabolic syndrome, obesity III, prediabetes, hyperinsulinemia, insulin resistance, and vitamin D deficiency. However, the patient had a long history of oral metformin therapy for type 2 diabetes mellitus; therefore, we still considered the patient to have T2DM. Abdominal ultrasonography revealed multiple gallstones. After MDT assessment, the patient was considered to meet the indications for metabolic and bariatric surgery, with no surgical contraindications. Given her young age and BMI below the threshold for super obesity (BMI >50 kg/m^2^), SILSG-JJB was selected, with concomitant cholecystectomy. Because this patient underwent concomitant cholecystectomy, the operative time was prolonged and intraoperative blood loss was greater than that of the previous two patients; however, no substantial differences were observed in postoperative exhaust time or length of postoperative hospital stay (Table 2). The patient was discharged on postoperative day 3 (Figure 2c), with a daily oral fluid intake exceeding 2,000 mL. At the 3-month follow-up, no surgery-related complications or malnutrition were observed. However, the patient experienced transient constipation in January, which was considered possibly caused by insufficient water intake. (Supplementary Tables S1, S2). Owing to postoperative supplementation with isolated whey protein and multivitamin chewable tablets, the patient's nutritional status improved compared with the preoperative condition. In addition, short-term glycemic parameters also showed improvement. Her body weight was reduced from 120 kg preoperatively to 107.5 kg at 1 month and 97.0 kg at 3 months postoperatively, resulting in improvements in %TBWL from 10.4% to 19.2% and %EWL from 23.7% to 43.6% (Table 3).

All three patients demonstrated excellent adherence to postoperative recommendations, including compliance with dietary instructions and supplementation with whey protein isolates and nutrient-dense chewable tablets. According to the *Chinese Guidelines for the Clinical Management of Obesity (2024 Edition)*, oral proton pump inhibitors and ursodeoxycholic acid are still required after surgery to prevent the occurrence of anastomotic ulcer and cholelithiasis. During the short-term follow-up at 3 months postoperatively, no umbilical wound-related complications were observed, including infection, pain, or incisional hernia. All patients reported high satisfaction with the cosmetic outcome and wound healing. Furthermore, progressive weight loss, improved glycemic control, and amelioration of metabolic comorbidities contributed to a further enhancement in overall quality of life. The three obese patients in this case series will continue to be followed at 6 months, 1 year, 2 years, 5 years, and beyond to further evaluate long-term outcomes and safety.

## 3 Patient selection

All three obese patients in this case series underwent SILSG-JJB. The selection of bariatric procedures at our center was primarily based on the *Chinese Clinical Guidelines for the Surgery of Obesity and Metabolic Disorders (2024 Edition)*. For patients with preoperatively confirmed gastroesophageal reflux disease (GERD), SG or SG-JJB is generally not recommended, whereas SG-TB or RYGB is preferred. In addition, RYGB is not recommended for patients with severe gastritis, gastric polyps, gastric ulcers, or a family history of gastric cancer identified during preoperative gastroduodenoscopy. Except for the above circumstances, patients shall make their own choice on the basis of fully understanding various current weight loss surgical procedures. It is oteworthy that our bariatric center is located in a region with a high incidence of gastric cancer. Since the excluded gastric remnant after RYGB is difficult to evaluate endoscopically, patients in this region generally have a low acceptance rate of RYGB.

Furthermore, for young obese patients who met the indications outlined in the *Chinese Expert Consensus on Single-Incision Laparoscopic Sleeve Gastrectomy (2025 Edition)*, both the advantages and potential disadvantages of single-incision and multiport laparoscopic approaches, as well as possible postoperative outcomes, were thoroughly explained preoperatively, after which the surgical approach was selected according to the patient's preference.

## 4 Discussion

### 4.1 The rationale for SG-JJB

Sleeve gastrectomy plus jejunojejunal bypass (SG-JJB), first introduced in 2003 (9), has gradually emerged as an important metabolic and bariatric surgical procedure. Liang et al. (10) reported postoperative outcomes in 244 Chinese patients with obesity and demonstrated that, in patients with a BMI ≥35 kg/m^2^, SG-JJB achieved superior weight loss compared with sleeve gastrectomy (SG) alone and comparable results to Roux-en-Y gastric bypass (RYGB). Moreover, SG-JJB was associated with fewer nutritional deficiencies and postoperative discomfort than RYGB (11). SG-JJB preserves pyloric function and avoids duodenal exclusion, thereby reducing the risk of dumping syndrome. In addition, compared with RYGB, SG-JJB is technically less complex and does not result in an excluded gastric remnant that is difficult to access endoscopically. By bypassing the proximal jejunum, SG-JJB reduces caloric absorption and promotes weight loss (12). More importantly, early delivery of nutrients to the distal ileum enhances incretin secretion, particularly from ileal L cells, leading to increased glucagon-like peptide-1 (GLP-1) release. GLP-1 has been shown to reduce *α*- and *β*-cell apoptosis, suppress glucagon secretion, and, together with peptide YY (PYY), enhance satiety and delay gastric emptying (13). These mechanisms collectively contribute to the improvement or remission of T2DM. Sepúlveda et al. (14) reported comparable T2DM remission rates between SG-JJB and RYGB at both 1- and 3-year follow-up. Taken together with our institutional experience, SG-JJB appears to provide superior short-term weight loss compared with SG alone in patients with BMI ≥40 kg/m^2^ and concomitant T2DM (15), while achieving glycemic outcomes comparable to RYGB, with fewer nutritional complications and lower technical complexity. Therefore, SG-JJB may represent a favorable surgical option in this specific patient population.

However, the presence of a jejunal blind loop after SG-JJB may carry a potential risk of blind loop syndrome. In the present case series, no procedure-related complications, including anastomotic bleeding, anastomotic leakage, foul-smelling flatus, or diarrhea, were observed during the 3-month follow-up period; nevertheless, these complications warrant close monitoring in subsequent long-term follow-up (Supplementary Table S1). Liang et al. reported that, during short-term follow-up after SG-JJB, no liver injury, infection, or fulminant diarrhea associated with bacterial overgrowth or bacterial translocation was observed following this procedure (10). The study also suggested that, in cases of severe symptoms caused by bacterial overgrowth, bleeding, or intestinal fistula, intestinal continuity could be readily restored. Furthermore, another study preliminarily demonstrated that SG-JJB was not associated with intestinal bacterial overgrowth, histopathological abnormalities, or signs of chronic infection after surgery (16). Nevertheless, further studies involving the Chinese obese population are still required to clarify whether blind loop syndrome occurs after SG-JJB and whether optimization of the anastomotic design could further reduce the incidence of these complications.

### 4.2 The rationale for the single-incision approach

**Figure 3:**
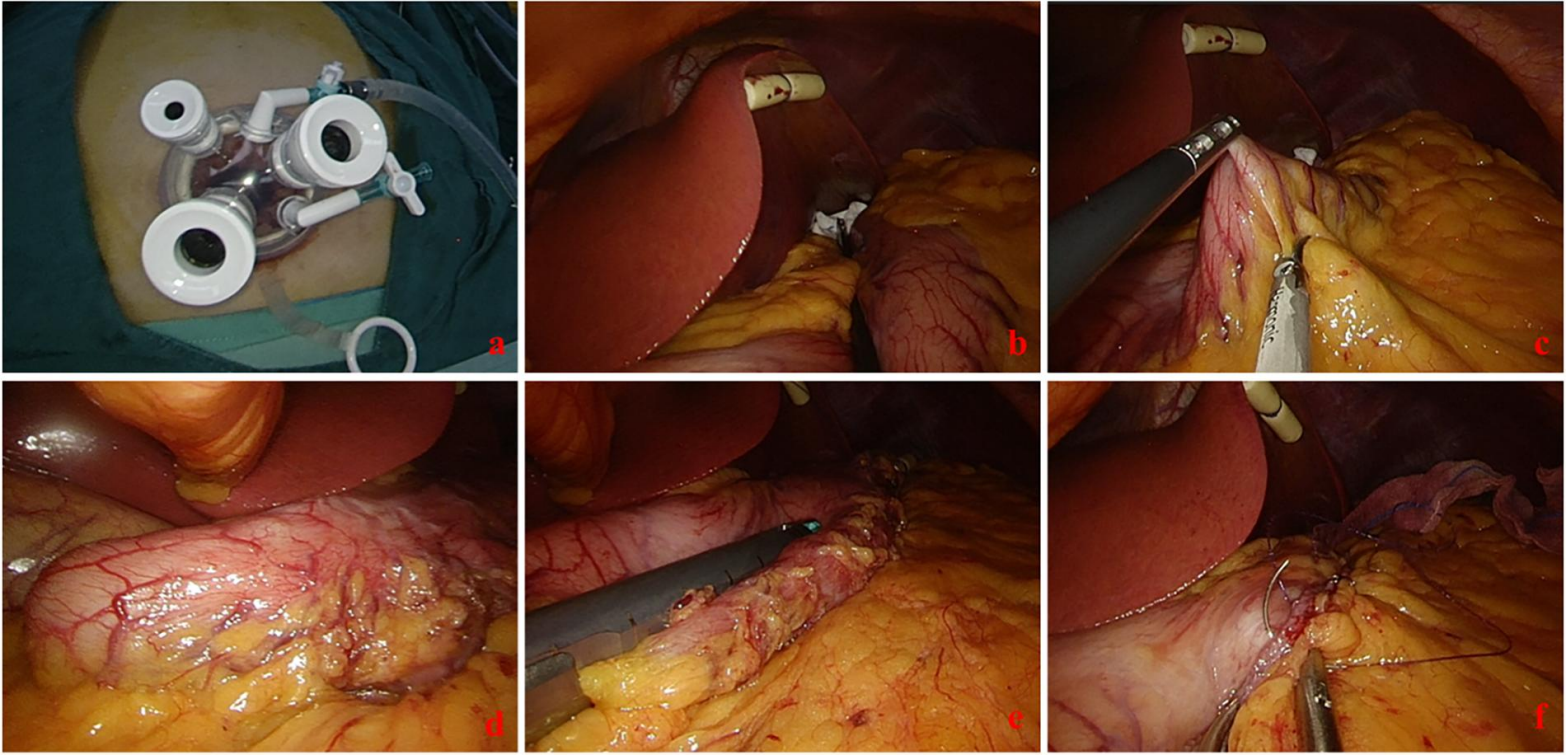
SILSG. **(a)** A single-incision trocar system was utilized during the operation. **(b)** The liver was retracted using a device fashioned from a T-tube and a purse-string suture. **(c)** Complete mobilization of the greater curvature extending to the gastric fundus was performed. **(d)** A 36-French bougie was inserted for calibration. **(e)** The stomach was transected along the greater curvature over a 36-French bougie. At our center, a 60 mm powered endoscopic linear stapler is routinely used. Green cartridges were applied for the first two firings, followed by blue cartridges for the remaining transections (blue cartridge: staple leg height 3.6 mm, closed staple height 1.5 mm; green cartridge: staple leg height 4.1 mm, closed staple height 2.0 mm). **(f)** The staple line of the remnant stomach was reinforced using continuous barbed sutures. A purse-string suture was placed at the gastric fundus. Approximately the proximal half of the staple line was further reinforced with seromuscular embedding sutures, while the distal half was continuously covered and reinforced by suturing the gastric remnant to the greater omentum. It was extracted from the surgical video of Case 3.

**Figure 4:**
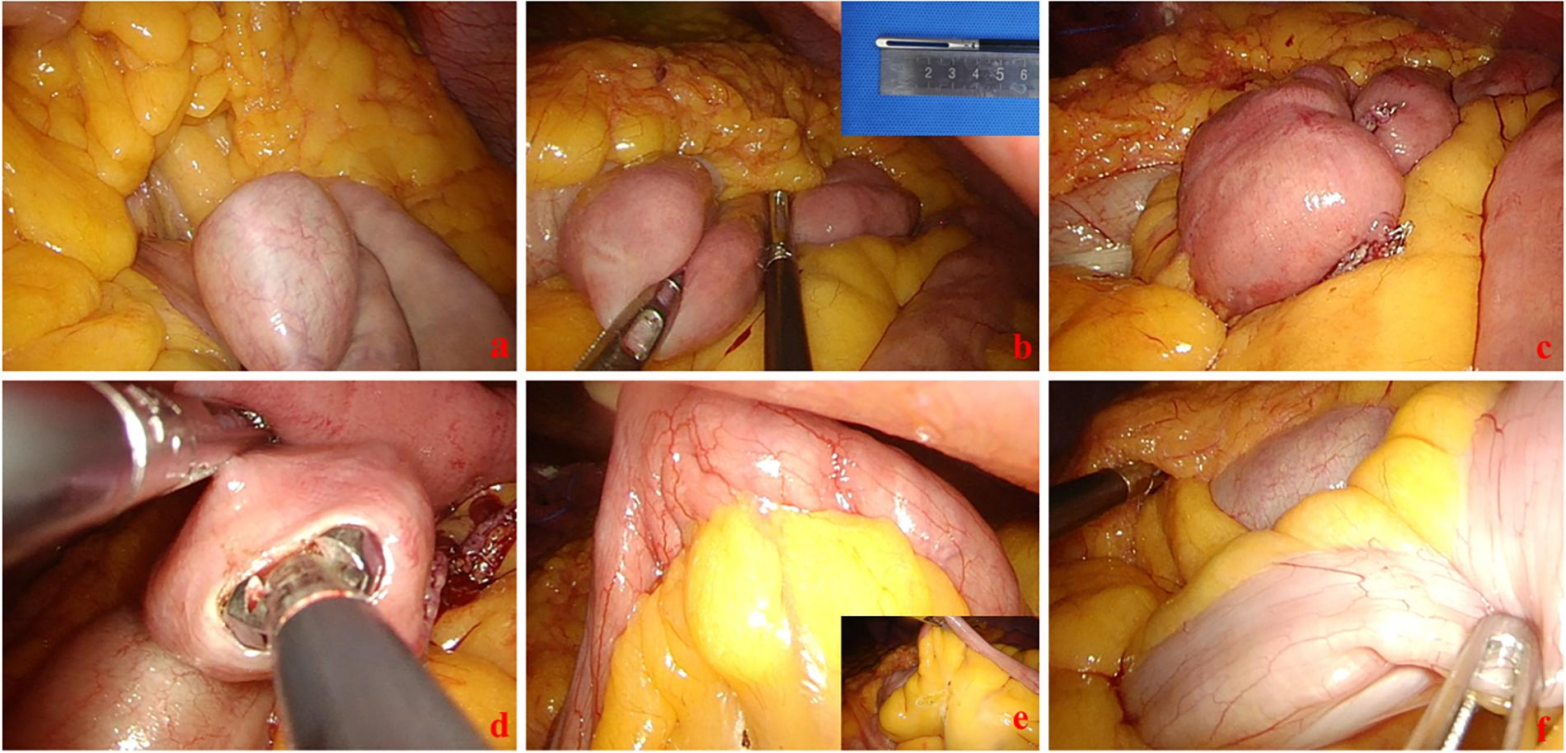
Jejunojejunal bypass **(a)** identification of the proximal small intestine. **(b)** Measurement of the small bowel using a 5 cm atraumatic clamp in a hand-over-hand manner. **(c)** Transection was performed approximately 30 cm distal to the beginning of the jejunum using a 60 mm powered endoscopic linear stapler with white cartridge (staple leg height 2.6 mm; closed staple height 1.0 mm). **(d)** An enterotomy was created on the proximal end of the transected bowel. **(e)** Further measurement of the small bowel to 300 cm, suspension of the bowel, and marking of the proximal and distal ends. **(f)** Continued distal measurement of the small bowel to the ileocecal junction. It was extracted from the surgical video of Case 3.

**Figure 5:**
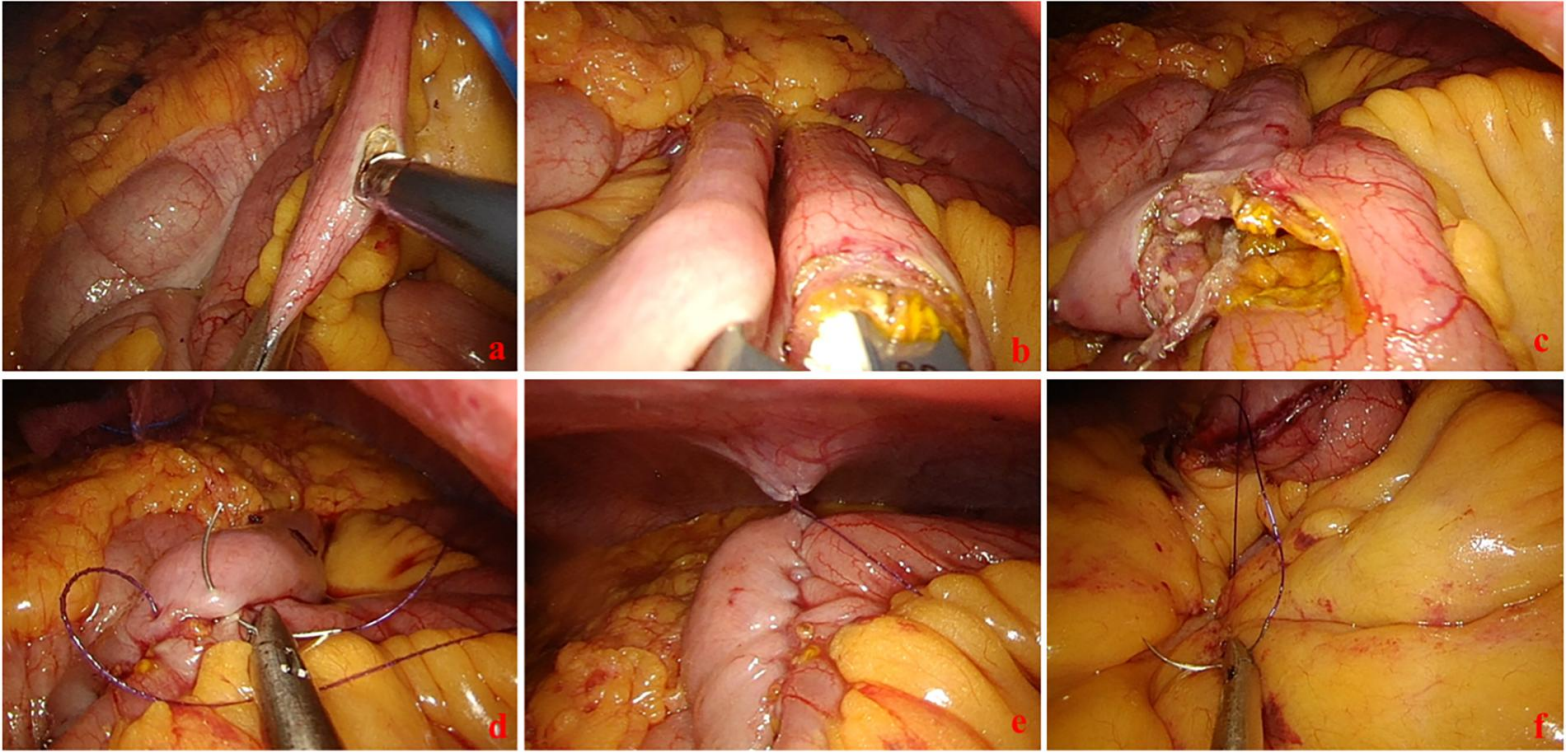
Jejunojejunal bypass **(a)** creation of an enterotomy at 300 cm of the small intestine. **(b)** Side-to-side anastomosis between the proximal jejunum at 30 cm and the distal small intestine at 300 cm. Our center continues to perform side-to-side intestinal anastomosis using a 60 mm powered endoscopic linear stapler with a white cartridge. In Case 1, the length of the bypassed jejunum was 300 cm, the length of the distal small intestine beyond the anastomosis was 260 cm, and the total small bowel length was 590 cm. In Case 2, the bypassed jejunal length was 300 cm, the distal small intestine beyond the anastomosis measured 285 cm, and the total small bowel length was 615 cm. In Case 3, the length of the bypassed jejunum was 300 cm, the distal intestinal limb beyond the anastomosis was 255 cm, and the total small bowel length was 585 cm. **(c)** Jejunojejunostomy. **(d)** Full-thickness closure of the anastomosis with barbed sutures, with seromuscular imbrication. **(e)** Suspension of the anastomosis to the abdominal wall using the remaining suture. **(f)** Closure of the mesenteric defect. It was extracted from the surgical video of Case 3.

SILSG-JJB is performed entirely through a concealed 2.0–2.5 cm transumbilical incision. The use of a single small incision may effectively reduce injury to the abdominal wall muscles, vessels, and nerves, thereby decreasing postoperative pain and improving patient comfort and cosmetic satisfaction (17). However, the implementation of SILSG-JJB is technically demanding due to limited operating space, instrument crowding, the unique anatomical characteristics of patients with obesity, and the high requirement for team coordination. To date, clinical reports specifically addressing SILSG-JJB remain scarce. Evidence from studies on SILSG suggests that single-incision and multi-port approaches do not differ significantly in terms of postoperative complications, including bleeding, wound infection, incisional hernia, or gastric leakage, nor in weight-loss outcomes and improvement of obesity-related comorbidities (18). Although single-incision procedures are often associated with longer operative times (19), this limitation can be mitigated through structured training and accumulated surgical experience. The key to successful single-incision laparoscopic surgery lies in the surgeon's adaptation to single-port ergonomics and effective exposure of the operative field. Based on our experience with single-incision laparoscopic sleeve gastrectomy (Figure 3), we gradually extended this technique to single-incision SG-JJB (Figures 4, 5), achieving safe and reproducible surgical outcomes in a selected patient population.

### 4.3 The specific technical considerations of SILSG-JJB

At present, the average operative time for SILSG performed by our team is less than 60 min. Compared with SILSG, SILSG-JJB is technically more demanding, as it requires additional procedures including accurate measurement of small bowel length, jejunojejunal side-to-side anastomosis, and subsequent closure of the mesenteric defect. During bowel measurement, placement of the single- incision device in the mid-abdomen provides more ergonomic access and facilitates *in situ* measurement of the small intestine with minimal traction. A 5 cm atraumatic bowel clamp was used to measure the small intestine in a hand-over-hand fashion from the ligament of Treitz to the terminal ileum (Figures 4a,b,f). Although previous studies have reported that this technique may be associated with measurement errors exceeding 15%, the margin of error can be substantially reduced through extensive laparoscopic training and the implementation of standardized surgical procedures (20, 21). After measuring 30 cm of jejunum, the bowel was transected, and an enterotomy was created on the antimesenteric side of the proximal limb (Figures 4c,d). When the small intestine was measured to 300 cm distally, the segment was marked to distinguish the proximal and distal limbs, and the jejunum was suspended using a seromuscular suture (Figure 4e). This strategy prevented loss of orientation during subsequent bowel manipulation and eliminated the need for repeated measurements, thereby reducing unnecessary traction injury. During closure of the mesenteric defect, the remaining suture used for closure of the jejunojejunal anastomosis was utilized to suspend the anastomosis to the abdominal wall (Figure 5e), which provided improved exposure of the mesenteric defect and facilitated safe closure (Figure 5f). As both the jejunojejunal anastomosis and the mesenteric defect were located in the left mid-abdomen, the single-incision approach avoided the unfavorable instrument angles commonly encountered in conventional multi-port layouts. This configuration may reduce traction-related injury to the bowel and mesentery, as well as the potential risk of anastomotic leakage, while improving suturing efficiency. In patients with obesity, thick abdominal walls and abundant subcutaneous fat can make umbilical closure technically challenging. To facilitate safe fascial closure and reduce the risk of postoperative umbilical hernia, the fascia and peritoneum were tagged with stay sutures at the time of incision, allowing for more efficient and reliable closure at the end of the procedure. The successful implementation of SILSG-JJB requires substantial experience in single-incision laparoscopic surgery and close cooperation between the primary surgeon and the camera operator. This case series was derived from a single-center cohort with a limited sample size and relatively short follow-up duration; therefore, it could only demonstrate the short-term efficacy and short-term postoperative complications of SILSG-JJB. At present, long-term follow-up data on SILSG-JJB remain limited, and further studies with larger cohorts and extended follow-up are warranted to validate its long-term safety and efficacy.

## 5 Conclusion

This case series demonstrates the technical feasibility and short-term safety of SILSG-JJB in selected patients with severe obesity and T2DM. During the short-term follow-up period, SILSG-JJB achieved satisfactory weight reduction and improvement in metabolic comorbidity-related parameters, including glycemic control, without procedure-related adverse events or postoperative malnutrition.

Accumulating evidence has suggested that single-incision laparoscopic surgery is a safe and feasible minimally invasive approach in metabolic and bariatric procedures. The single-incision approach may provide potential advantages, including reduced surgical trauma, improved cosmetic outcomes, and enhanced patient satisfaction, while remaining consistent with the principles of enhanced recovery after bariatric surgery.

However, the wider adoption of SILSG-JJB remains limited by technical complexity, the lack of standardized training pathways, and the current paucity of high-level evidence. Furthermore, given the limited sample size and short follow-up duration in the present study, the long-term efficacy and safety of SILSG-JJB in obese patients with T2DM and other metabolic complications require further investigation. Future multicenter prospective studies with larger cohorts and longer follow-up periods are necessary to evaluate long-term metabolic outcomes, nutritional status, postoperative complications, safety, and generalizability, and may facilitate the standardized implementation of SILSG-JJB in more bariatric centers.

## Data Availability

The original contributions presented in the study are included in the article/Supplementary Material, further inquiries can be directed to the corresponding author/s.
