# Supplementary material for: Case Report: Single-incision laparoscopic sleeve gastrectomy plus jejunojejunal bypass for the treatment of type 2 diabetes in patients with obesity: a case series and review
Source: Front Surg. 2026 Jun 11;13:1855934. doi: 10.3389/fsurg.2026.1855934 (PMC13293643; doi:10.3389/fsurg.2026.1855934)
Supplement: Supplementary file 1 [file Table1.docx]

**Supplement Table 1. Postoperative Complications During Follow-up**

| **Complication** | **1 Month (n=3)** | **3 Months (n=3)** |
| --- | --- | --- |
| **GERD** | 0 | 0 |
| **Anemia** | 0 | 0 |
| **Anastomotic Leak** | 0 | 0 |
| **Hypoproteinemia** | 0 | 0 |
| **Vitamin D deficiency** | 1，Case2 | 1，Case2 |
| **Vitamin B12 deficiency** | 0 | 0 |
| **pulmonary infection** | 0 | 0 |
| **Bleeding** | 0 | 0 |
| **Incomplete intestinal obstruction** | 0 | 0 |
| **Intussusception** | 0 | 0 |
| **Constipation** | 1，Case3 | 0 |
| **Malodorous flatus** | 0 | 0 |
| **Diarrhea** | 0 | 0 |
