# Supplementary material for: Case Report: Single-incision laparoscopic sleeve gastrectomy plus jejunojejunal bypass for the treatment of type 2 diabetes in patients with obesity: a case series and review
Source: Front Surg. 2026 Jun 11;13:1855934. doi: 10.3389/fsurg.2026.1855934 (PMC13293643; doi:10.3389/fsurg.2026.1855934)
Supplement: Supplementary file 2 [file Table2.docx]

**Supplement Table 2. Laboratory Parameters During Follow-up**

| **Variable** | **Case 1** | **Case 2** | **Case 3** | **Reference range** | **Unit** |
| --- | --- | --- | --- | --- | --- |
| **1 month after surgery** | | | | | |
| **Albumin** | 44.4(N) | 45.6(N) | 44.7(N) | 40.0-55.0 | g/L |
| **FPG** | 4.02(N) | 5.87(N) | 5.44(N) | 3.9-6.1 | mmol/L |
| **Potassium** | 3.36(N) | 3.74(N) | 4.39(N) | 3.5-5.3 | mmol/L |
| **TC** | 4.47(N) | 3.25(N) | 4.69(N) | 2.3–5.2 | mmol/L |
| **TG** | 1.21(N) | 1.28(N) | 1.49(N) | 0.56–1.70 | mmol/L |
| **LDL-C** | 2.94(N) | 2.03(N) | 3.18(N) | 1.20–3.30 | mmol/L |
| **UA** | 646.0(H) | 637.0(H) | 352.0(N) | 155–357 | μmol/L |
| **25(OH)Vit D** | 38.50(N) | 16.50 (L) | 30.90(N) | 30–100 | ng/mL |
| **Vitamin B12** | 709.00(N) | 1980.00(H) | 1755.00(H) | 179-771 | Pg/mL |
| **3 months after surgery** | | | | | |
| **Albumin** | 41.8(N) | 43.3(N) | 47.4(N) | 40.0-55.0 | g/L |
| **FPG** | 4.81(N) | 5.42(N) | 5.11(N) | 3.9-6.1 | mmol/L |
| **Potassium** | 3.47(N) | 3.88(N) | 4.57(N) | 3.5-5.3 | mmol/L |
| **TC** | 4.87(N) | 3.49(N) | 3.75(N) | 2.3–5.2 | mmol/L |
| **TG** | 0.96(N) | 1.15(N) | 1.01(N) | 0.56–1.70 | mmol/L |
| **LDL-C** | 3.21(N) | 2.17(N) | 2.27(N) | 1.20–3.30 | mmol/L |
| **UA** | 402.0(H) | 541.8(H) | 403.0(H) | 155–357 | μmol/L |
| **25(OH)Vit D** | 50.60(N) | 22.40 (L) | 41.50(N) | 30–100 | ng/mL |
| **Vitamin B12** | 682.00(N) | 2000.00(H) | 2000.00(H) | 179-771 | Pg/mL |

N, normal; H, high; L, low.
